# The effects of magnesium-zinc-calcium-vitamin D co-supplementation on biomarkers of inflammation, oxidative stress and pregnancy outcomes in gestational diabetes

**DOI:** 10.1186/s12884-019-2258-y

**Published:** 2019-03-29

**Authors:** Mehri Jamilian, Naghmeh Mirhosseini, Masoumeh Eslahi, Fereshteh Bahmani, Maryam Shokrpour, Maryam Chamani, Zatollah Asemi

**Affiliations:** 1 Traditional and Complementary Medicine Research Center, Arak University of Medical Sciences, Arak, Iran; 2 School of Public Health, University of Saskatchewan, Saskatoon, SK Canada; 3 Research Center for Biochemistry and Nutrition in Metabolic Diseases, Kashan University of Medical Sciences, Kashan, IR Iran; 4 Department of Gynecology and Obstetrics, School of Medicine, Iran University of Medical Sciences, Tehran, Iran

**Keywords:** Supplementation, Multinutrients, Gestational diabetes, Pregnancy, Inflammation

## Abstract

**Background:**

Diabetes is the most common medical condition in pregnant women and its complications affect both mother and fetus. The beneficial effects of vitamin D on gestational diabetes have been shown, though data on the effects of co-administration of vitamin D with other nutrients on pregnancy outcomes in gestational diabetes (GDM) are scarce. This study was aimed to determine the effects of magnesium-zinc-calcium-vitamin D co-supplementation on parameters of inflammation and oxidative stress, and pregnancy outcomes among women with GDM.

**Methods:**

This randomized, double-blinded, placebo-controlled trial was conducted on 60 women with GDM not taking oral hypoglycemic agents. Patients were randomly assigned to take magnesium-zinc-calcium-vitamin D supplements (*n* = 30) or placebo (*n* = 30) for 6 weeks. Fasting blood samples were collected from participants at baseline and after the 6-week intervention to measure related biomarkers.

**Results:**

Magnesium-zinc-calcium-vitamin D co-supplementation resulted in a significant reduction in serum high-sensitivity C-reactive protein (− 1.2 ± 3.5 vs. + 0.8 ± 2.0 mg/L, *P* = 0.01) and plasma malondialdehyde concentrations (− 0.3 ± 0.3 vs. + 0.3 ± 1.1 μmol/L, *P* = 0.003), as well as a significant increase in total antioxidant capacity levels (+ 38.2 ± 76.5 vs. -16.3 ± 93.5 mmol/L, P = 0.01), compared to placebo. We found a decreasing trend in newborns’ weight (3089.8 ± 519.9 vs. 3346.3 ± 411.1 g, *P* = 0.05) and the rate of macrosomia (3.3% vs. 16.7%, *P* = 0.08) in the magnesium-zinc-calcium-vitamin D supplemented women.

**Conclusions:**

Overall, the findings of this study have demonstrated that magnesium-zinc-calcium-vitamin D co-supplementation for 6 weeks to women with GDM may reduce biomarkers of inflammation and oxidative stress.

This study was retrospectively registered on 25 April 2017 in the Iranian website (www.irct.ir) for clinical trials registration (http://www.irct.ir: IRCT201704225623N109).

## Background

Gestational diabetes mellitus (GDM) is defined as a state of glucose intolerance that is diagnosed for the first time during pregnancy [1]. Multiple risk factors, including gestational age, obesity, ethnic background, family history of type 2 diabetes (T2DM) and a prior history of GDM have been implicated as the contributors to GDM [2]. The prevalence of GDM was reported to rates as high as 15–20% worldwide [3]. In Iran, the prevalence has been as high as 18.6% [4]. Extra calories and increased weight during pregnancy induce inflammatory pathway among GDM women, which in turn would result in the development of insulin resistance and excessive fetal growth; macrosomia [5]. Moreover, there are evidence suggesting that underlying factors like obesity enforce oxidative stress; increased oxidative damage and reduced antioxidant capacity, in women with GDM, which contributes to the initiation and progression of GDM [6, 7].

Serum levels of magnesium, zinc, calcium and vitamin D have been reported to be significantly lower in women with GDM compared to healthy pregnant women [8, 9]. The beneficial effects of single-nutrient supplementation including magnesium [10], zinc [11], calcium and its co-administration with vitamin D [12] on attenuating inflammation and oxidative stress, and improving pregnancy outcomes have been proved in women with GDM. However, to our best knowledge the effects of multinutrients supplementation (magnesium-zinc-calcium-vitamin D) on inflammation and oxidative stress markers and pregnancy outcomes have not been assessed in women with GDM yet. The synergist immunomodulatory, anti-inflammatory and antioxidant effects of magnesium, zinc, calcium and vitamin D [13, 14] might enhance their impact on pregnancy outcomes in women with GDM. Same research team already published that calcium and vitamin D co-supplementation for 8 weeks diminished inflammation and oxidative stress markers in women with polycystic ovary syndrome (PCOS). These effects were more highlighted rather than supplementing these women with calcium or vitamin D alone [15].

There are promising evidence suggesting the importance of different nutrients (magnesium, zinc, calcium, vitamin D) on inflammation and oxidative stress markers and pregnancy outcomes in women with GDM. Therefore, we designed this placebo-controlled trial to determine the synergistic effects of magnesium-zinc-calcium-vitamin D co-supplementation on inflammation and oxidative stress and subsequent pregnancy outcomes among women with GDM who were not on oral hypoglycemic agents.

## Methods

### Ethics statements

This study was approved by the Ethics Committee of the Arak University of Medical Sciences (AUMS). It was conducted according to the principles explained in the Declaration of Helsinki. All participants provided written and informed consent. The present clinical trial was registered at the Iranian website for registration of clinical trials (www.irct.ir: IRCT201704225623N109).

### Study design and population

This was a prospective, randomized, double-blind, placebo-controlled trial of women with GDM. This single-center study was conducted in Arak, Iran between March and November 2017. Participants were 60 women, aged 18 to 40 years, with GDM who were referred to the Kosar Clinic in Arak, Iran. Gestational diabetes was diagnosed using a “one-step” 2-h 75-g oral glucose tolerance test (OGTT) at 24–28 weeks’ gestation and was applied as the inclusion criteria for this trial. Diagnosis of GDM was based on the American Diabetes Association guidelines [16]: women whose plasma glucose level met one of the following criteria were considered as having GDM: fasting plasma glucose level (FPG) ≥ 92 mg/dL, 1 h OGTT≥180 mg/dL and 2 h OGTT≥153 mg/dL. Exclusion criteria included: history of placenta abruption, pre-eclampsia, eclampsia, hypo and hyperthyroidism, kidney or liver diseases, taking magnesium, zinc, calcium and vitamin D supplements prior to intervention, being smoker, and insulin therapy after GDM diagnosis.

### Randomization and treatment allocation

At the baseline visit, 60 women with GDM were randomly assigned to receive either 100 mg magnesium, 4 mg zinc, 400 mg calcium plus 200 IU vitamin D supplements (*n* = 30) or placebo (n = 30) twice a day for 6 weeks. After the termination of intervention at the end of week 6, all women were monitored for pregnancy outcomes until the delivery. Supplements and placebos tablets had identical shape and size, and manufactured by Vitane (Wolfratshausen, Germany) and Barij Essence Pharmaceuticals (Kashan, Iran), respectively. All study participants followed the standard pregnancy protocol in Iran, consuming 1000 IU vitamin D3 and 400 μg/day vitamin B9, from the beginning of pregnancy, and 60 mg/day ferrous sulfate, from the second trimester. Patients were requested to come back in order to check their blood glucose levels weekly (besides their daily self-monitoring) during the study. In order to improve the compliance rate, participants received a short message on their cell phones as a reminder for supplement consumption daily. To evaluate the compliance rate, subjects were asked to bring back the supplement container at the following visits. To determine the compliance, the remaining supplements were counted and subtracted from the amount of supplements provided to the participants. Moreover, serum magnesium, zinc, calcium and vitamin D levels were measured to more assess the compliance rate to multinutrients supplementation. Study participants were randomized using computer-generated random numbers. Randomization and allocation were concealed from the researchers and participants until the final analyses were completed. The randomized allocation sequence, enrolling participants and allocating them to intervention groups were conducted by a trained midwife at the gynecology clinic. Another person, who was not involved in the trial and not aware of random sequences, assigned the subjects to the numbered bottles of capsules. All participants were requested to maintain their routine dietary habits and physical activity during the trial. All women completed a 3-day food record and three physical activity records presented as metabolic equivalents at weeks 0, 3 and 6 of the intervention.

### Outcomes

In the present study, the primary outcomes were serum hs-CRP and plasma total nitrite levels. The secondary outcomes were the biomarkers of oxidative stress and pregnancy outcomes.

### Anthropometric measures

Anthropometric measurements were conducted by a trained midwife at baseline and the end of the intervention. Weight and height were measured using a Seca 713 scale without shoes and in light clothing to the nearest 0.1 kg and 0.1 cm, respectively. BMI was calculated as the ratio of the current body weight to height squared (kg/m^2^). After labor, all newborns’ weight and length were measured by a trained midwife using the standard methods (Seca 155 Scale, Hamburg; Germany). Infants’ head circumference was measured to the nearest 1 mm with a Seca girth measuring tape. Moreover, we determined infants’ 1- and 5-min Apgar scores as another measure of pregnancy outcome.

### Clinical measures

Polyhydramnios was diagnosed using the sonographic estimation method at the end of the trial. Applying sonography, polyhydramnios was detected when amniotic fluid index (AFI) exceeded 25 cm [17]. Preterm delivery was defined as delivery occurred at < 37 weeks of pregnancy and newborn’s macrosomia was defined as baby’s birth weight of > 4000 g. Large-for-gestational-age (LGA) newborns were live-born infants with their birth weight ≥ 90th percentile of birth weight according to the latest normograms based on gender and gestational age [18].

### Biochemical measures

Ten milliliter fasting blood was collected for biomarkers measurements at weeks 0 and 6 of the intervention. Serum magnesium, zinc and calcium concentrations were measured using enzymatic kits (Pars Azmun, Tehran, Iran), with inter- and intra-assay coefficient variances (CVs) of less than 5%. To determine FPG, we used enzymatic kits (Pars Azmun, Tehran, Iran). Serum 25-hydroxyvitamin D concentrations were measured using a commercial ELISA kit (IDS, Boldon, UK) with inter- and intra-assay CVs of 4.6 and 6.4%, respectively. Serum hs-CRP levels were assessed using ELISA kit (LDN, Nordhorn, Germany) with intra- and inter-assay CVs of 4.0 and 6.1%, respectively. Part of blood samples were immediately centrifuged (3000×g, 10 min, 4^°C^) after collection; the plasma was then separated and stored at -70^oC^ until the analysis for total nitrite, malondialdehyde (MDA), total antioxidant capacity (TAC), and GSH. The plasma total nitrite concentrations were measured using Griess method [19]; GSH concentrations by the method of Beutler et al. [20] and MDA levels using thiobarbituric acid reactive substance spectrophotometric test [21]. Plasma TAC concentrations were measured using the ferric reduction antioxidant power method developed by Benzie and Strain [22]. CVs for plasma total nitrite, TAC, GSH and MDA were lower than 5%. Newborns’ hyperbilirubinemia was determined by the total serum bilirubin levels above 15 mg/dL (257 μmol/L) among infants who were 25 to 48 h old, 18 mg/dL (308 μmol/L) in infants who were 49 to 72 h old, and above 20 mg/dL (342 μmol/L) in infants older than 72 h [23].

### Sample size calculation

Applying hs-CRP as a primary outcome with a mean distinction of 3.2 mg/L and a SD of 4.0 mg/L, we used the standard formula for sample size calculation in clinical trials. Considering a type one error (α) of 0.05 and type two error (β) of 0.20 with the power of 80%, the calculated sample size was 25 subjects in each treatment group [24]. Assuming 5 dropouts in each group, the final sample size was determined to be 30 subjects in each group.

### Statistical analysis

Kolmogorov-Smirnov test was used to check the normal distribution of data. General characteristics and dietary intakes were compared between two treatment groups using an independent-samples *t*-test. To determine the effects of magnesium-zinc-calcium-vitamin D on biomarkers of inflammation and oxidative stress, we used independent-samples *t*-test. To control confounding variables including; baseline values, maternal age and baseline BMI, we used ANCOVA test, using general linear models. Differences in proportions were assessed using Chi square test or Fisher’s exact tests. *P* < 0.05 was considered as statistically significant. All statistical analyses were conducted using the SPSS Software (version 18.0, SPSS Inc., Chicago, Illinois, USA).

## Results

**Fig. 1:**
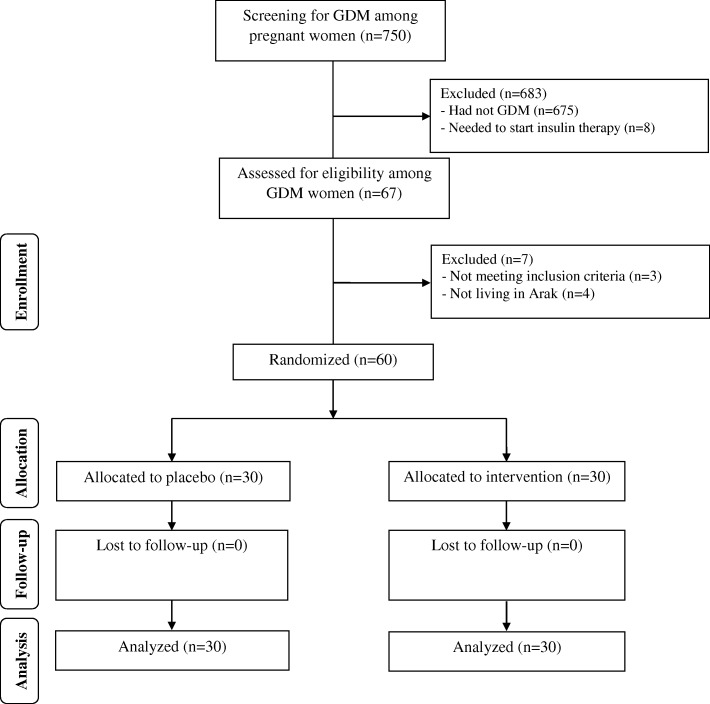
Summary of patient flow diagram

The flow of participants’ recruitment has been demonstrated in Fig. 1. Sixty participants completed the trial, *n* = 30 in magnesium-zinc-calcium-vitamin D arm and n = 30 in placebo arm. Sending daily reminders for supplements consumption, relying on participants’ responds and by considering that higher than 90% of capsules were consumed throughout the trial in both groups, assessed through empty bottles back, we assume that the compliance rate in the current study was acceptable.

The mean age, height, weight and BMI, at both baseline and the end of the intervention, were not significantly different between magnesium-zinc-calcium-vitamin D and placebo groups (Table 1).

**Table 1:** General characteristics of study participants

	Placebo group (*n* = 30)	Magnesium-zinc-calcium-vitamin D group (*n* = 30)	P^1^
Age (y)	29.1 ± 4.1	27.7 ± 4.0	0.19
Height (cm)	163.5 ± 3.7	162.8 ± 4.1	0.48
Weight at study baseline (kg)	67.6 ± 6.1	68.2 ± 9.4	0.78
Weight at end-of-trial (kg)	69.4 ± 6.1	70.0 ± 9.2	0.76
BMI at study baseline (kg/m^2^)	25.3 ± 2.5	25.8 ± 3.7	0.66
BMI at end-of-trial (kg/m^2^)	26.0 ± 2.6	26.4 ± 3.6	0.58

Data are means± SDs

^1^Obtained from independent-samples *t*-test

Using the 3-day dietary records obtained at baseline, end-of-trial and throughout the trial, we observed no significant difference in macro- and micronutrient intakes between the two groups during the study (Data not shown).

Biomarkers of inflammation and oxidative stress at baseline and after the 6-wk intervention in women with GDM who received either magnesium-zinc-calcium-vitamin D or placebo are presented in Table 2. Compared with the placebo, co-supplementation with magnesium-zinc-calcium-vitamin D significantly increased serum magnesium (+ 0.1 ± 0.2 vs. -0.05 ± 0.1 mg/dL, *P* = 0.002), zinc (+ 4.1 ± 1.8 vs. + 0.4 ± 2.6 mg/dL, *P* < 0.001), calcium (+ 0.3 ± 0.4 vs. + 0.1 ± 0.1 mg/dL, *P* = 0.001) and 25-OH-vitamin D (+ 6.1 ± 3.5 vs. + 3.8 ± 1.2 ng/mL, P = 0.001). The co-supplementation of magnesium-zinc-calcium-vitamin D resulted in a significant reduction in FPG (− 4.3 ± 4.9 vs. − 0.9 ± 4.5 mg/dL, *P* = 0.008) and serum hs-CRP (− 1.2 ± 3.5 vs. + 0.8 ± 2.0 mg/L, *P* = 0.01) and plasma MDA concentrations (− 0.3 ± 0.3 vs. + 0.3 ± 1.1 μmol/L, *P* = 0.003), and a remarkable increase in TAC levels (+ 38.2 ± 76.5 vs. -16.3 ± 93.5 mmol/L, P = 0.01), compared to placebo**.**

**Table 2:** Biomarkers of inflammation and oxidative stress at baseline and after 6-wk intervention in women with gestational diabetes who received either magnesium-zinc-calcium-vitamin D or placebo

	Placebo group (*n* = 30)			Magnesium-zinc-calcium-vitamin D group (*n* = 30)			P^1^
	Wk0	Wk6	Change	Wk0	Wk6	Change	
FPG (mg/dL)	95.1 ± 5.4	94.2 ± 5.7	-0.9 ± 4.5	93.1 ± 2.5	88.8 ± 4.7	-4.3±4.9	0.008
Magnesium (mg/dL)	1.7 ± 0.2	1.7 ± 0.2	− 0.05 ± 0.1	1.8 ± 0.1	1.8 ± 0.2	0.1 ± 0.2	0.002
Zinc (mg/dL)	83.9 ± 8.1	84.3 ± 8.1	0.4 ± 2.6	80.3 ± 8.8	84.4 ± 9.1	4.1 ± 1.8	< 0.001
Calcium (mg/dL)	9.1 ± 0.5	9.2 ± 0.5	0.1 ± 0.1	9.1 ± 0.4	9.4 ± 0.4	0.3 ± 0.4	0.001
25-OH-vitamin D (ng/mL)	13.5 ± 3.6	17.3 ± 3.1	3.8 ± 1.2	12.6 ± 4.2	18.7 ± 4.7	6.1 ± 3.5	0.001
hs-CRP (mg/L)	6.3 ± 2.0	7.0 ± 2.6	0.8 ± 2.0	6.9 ± 1.7	5.7 ± 3.7	−1.2 ± 3.5	0.01
Total nitrite (μmol/L)	40.9 ± 10.3	41.2 ± 17.7	0.3 ± 15.3	43.1 ± 5.5	44.3 ± 6.1	1.2 ± 7.1	0.76
TAC (mmol/L)	694.8 ± 111.6	678.4 ± 89.9	−16.3 ± 93.5	619.4 ± 81.1	657.6 ± 98.1	38.2 ± 76.5	0.01
GSH (μmol/L)	475.9 ± 112.0	466.9 ± 109.2	−9.0 ± 96.6	474.2 ± 121.7	486.1 ± 115.6	11.9 ± 63.7	0.32
MDA (μmol/L)	3.0 ± 0.7	3.3 ± 1.3	0.3 ± 1.1	2.9 ± 0.3	2.6 ± 0.2	−0.3 ± 0.3	0.003

All values are means± SDs

^1^*P* values represent the time × group interaction (computed by analysis of the repeated measures ANOVA)

*FPG* fasting plasma glucose, *GDM* gestational diabetes mellitus, *GSH* total glutathione, *hs-CRP* high-sensitivity C-reactive protein, *MDA* malondialdehyde, *TAC* total antioxidant capacity

Newborns’ weight (3089.8 ± 519.9 vs. 3346.3 ± 411.1 g, *P* = 0.05) and the rate of macrosomia (3.3% vs. 16.7%, *P* = 0.08) was lower in the magnesium-zinc-calcium-vitamin D group compared to the placebo group (Table 3). However, we did not find a significant difference in the rate of insulin therapy after intervention, polyhydramnios and maternal hospitalization. Gestational age, newborn’s birth size and Apgar scores did not significantly change following co-supplementation of magnesium-zinc-calcium-vitamin D.

**Table 3:** The association between magnesium-zinc-calcium-vitamin D co-supplementation and pregnancy outcomes

	Placebo group (*n* = 30)	Magnesium-zinc-calcium-vitamin D group (*n* = 30)	P^1^
Cesarean section (%)	9 (30.0)	4 (13.3)	0.11^†^
Preterm delivery (%)	1 (3.3)	0 (0.0)	0.31^†^
Need to insulin therapy after intervention (%)	2 (6.7)	1 (3.3)	0.55^†^
Pre-eclampsia (%)	3 (10.0)	2 (6.7)	0.64^†^
Polyhydramnios (%)	2 (6.7)	1 (3.3)	0.55^†^
Macrosomia > 4000 g (%)	5 (16.7)	1 (3.3)	0.08^†^
Gestational age (weeks)	39.2 ± 1.1	39.1 ± 1.2	0.90
Newborns’ weight (g)	3346.3 ± 411.1	3089.8 ± 519.9	0.05
Newborns’ length (cm)	50.0 ± 1.4	49.2 ± 3.6	0.28
Newborns’ head circumference (cm)	35.2 ± 1.7	34.4 ± 3.0	0.20
1- min Apgar score	8.8 ± 0.4	8.9 ± 0.3	0.45
5- min Apgar score	9.8 ± 0.4	9.9 ± 0.3	0.45
Newborns’ hyperbilirubinemia (%)	12 (40.0)	8 (26.7)	0.27^†^
Newborns’ hospitalization (%)	12 (40.0)	8 (26.7)	0.27^†^

Values are means± SDs for continuous measures and are number (%) for dichotomous variables

^1^Obtained from independent *t*-test

^†^Obtained from Pearson Chi-square test

## Discussion

Gestational diabetes poses risks for both mother and fetus. Pregnant women with obesity or GDM are insulin-resistant compared to normal pregnant women, which equates to increased maternal inflammation [25]. Suppression of inflammation in these patients helps improving pregnancy outcomes and maternal complications [26]. Current evidence is suggesting that magnesium-zinc-calcium-vitamin D co-supplementation has beneficial effects on metabolic profiles of women with GDM, such as improving insulin sensitivity and few lipid profiles [27]. We assumed that the beneficial impacts of multi-nutrients therapy on metabolic parameters might be related to its attenuating effect on inflammatory markers which are the root causes of the metabolic abnormalities. The findings of this study have demonstrated that magnesium-zinc-calcium-vitamin D co-supplementation for 6 weeks to women with GDM may reduce biomarkers of inflammation and oxidative stress.

It must be kept in mind that all pregnant women in Iran have a monthly check-up meanwhile they are monitored for vitamin D supplements compliant and whether they have had any side effect with this supplement. In this study women were compliant with the standard protocol of pregnancy for vitamin D. Since vitamin D requirement is increased in pregnancy, we added 400 IU to the combination of other elements and we did not add more because of risk of side effects as well as we believe calcium might have synergistic effect with vitamin D. Observing significant effects in this study moreover clarifies that although 1000 + 400 IU/day vitamin D might not be much different from 1000 IU/day in placebo group, however combination of other helpful nutrients with vitamin D can differentiate the impact of 1000 + 400 IU/day from 1000 IU/day. However, in the current study, magnesium levels increased by 0.1 mg/dL in the intervention group, which was statistically significant although might not be clinically significant. The important point that should be considered is that serum magnesium concentrations do not thoroughly reflect dietary or supplemental magnesium intake. Although serum magnesium levels are dependent on dietary intake, due to difference in intestinal absorption and kidney function, urinary magnesium excretion and intracellular magnesium concentrations are better indicators for magnesium status than serum magnesium levels. These parameters are also more sensitive to oral supplementation than serum magnesium concentrations. However, we were not able to assess intracellular magnesium concentrations in the current study due to funding limitations. Some investigators have also recommended applying erythrocyte magnesium content to assess dietary intake [28]. Others have shown that the magnesium content of white blood cells is a better index of intracellular magnesium in skeletal and cardiac muscle [28]. Overall, due to low sensitivity of serum magnesium for assessing magnesium status, our results were not clinically significant.

### Effects on biomarkers of inflammation and oxidative stress

Elevated hs-CRP is more common among women with GDM as compared to women with normal pregnancy. A reference value of hs-CRP levels below 3 mg/L is considered normal [29], and there are a few studies have reported circulating levels of hs-CRP higher than 3 mg/L in women with GDM [30, 31]. Our findings demonstrated that co-administering of magnesium-zinc-calcium-vitamin D for 6 weeks to women with GDM attenuated inflammation and oxidative stress, through decreasing serum hs-CRP, plasma total nitrite, and MDA levels, and increasing TAC levels. The impact of mono-nutrient therapy has been depicted in already published clinical trials. We have previously shown that magnesium oxide (250 mg/day) plus zinc sulfate (220 mg/day) co-supplementation for 12 weeks had beneficial effects on hs-CRP and TAC levels, and gene expression related to IL-1 and TNF-α in women diagnosed with PCOS [32]. Consistent with our results, co-administration of calcium (1000 mg/day) with vitamin D (50,000 IU/week) supplements for 12 weeks has been shown to significantly lessen systemic inflammation, through decreasing IL-6 and TNF-α levels in patients with T2DM [33]. Moreover, among young men with sedentary lifestyle, magnesium supplementation for 4 weeks had remarkable impacts on diminishing DNA oxidative damage [34]. In another study, taking zinc gluconate by obese individuals, at a dosage of 30 mg/day, for 8 weeks resulted in a significant reduction in serum hs-CRP concentrations [35]. Elevated inflammatory cytokines and oxidative stress during pregnancy contribute to increased cardiovascular risk [36] and insulin resistance post-partum [31, 37]. Anti-inflammatory effects of magnesium, vitamin D and zinc may be due to their antagonistic impact on serum calcium [31, 38] and their role in regulating nuclear factor-κB activity, and peroxisome proliferator activated receptor-α signaling pathway, which require zinc and magnesium for their anti-inflammatory effects [39]. In addition, less production of parathyroid hormone following the intake of calcium and vitamin D supplements may result in the reduced production of inflammatory factors [40]. Magnesium and zinc intake might reduce oxidative damage through decreasing ROS production [41] and decrease the formation of ^·^OH from hydrogen peroxide by counteracting the redox-active transition metals [42]. Furthermore, antioxidant impact of vitamin D might be explained by a significant decrease in ROS and pro-inflammatory markers [43].

### Effects on pregnancy outcomes

Additional demands associated with pregnancy and fetal growth, pose pregnant women at more risk for multiple nutrient deficiencies [44]. The results of current study revealed a decreasing trend in newborns’ weight and the rate of macrosomia in the magnesium-zinc-calcium-vitamin D co-supplemented group compared to the placebo group. Following supplementation, the difference between birth weights was large and non-significant between intervention groups (3089.8 vs. 3346.3, *P* = 0.05). This result has been presented as mean values, however when we assessed data of birth weight individually we did not see any birth weight below normal value. On the other hand, the reduction in macrosomia rate was one of the goals of this study. Overall, based on current findings we did not consider these reductions as negative effects. Same research group has already shown that magnesium supplementation might decrease the incidence of newborn hyperbilirubinemia and newborn hospitalization and was less associated with macrosomia [10]. Looking at the effect of zinc on pregnancy outcomes, the results are inconclusive, which might be related to high incidence of zinc deficiency in pregnancy and low doses of supplementation are usually used in different trials [45, 46]. The combination of vitamin D and calcium has been shown significant reduction in the rate of macrosomia and caesarean section in women with GDM [12]. The inconclusive results in the current trial, as a multi-nutrients supplementation approach might be related to the following parameters; supplementation strategies might be beneficial when a deficiency is observed, maternal weight gain during pregnancy is of major importance and is affected by different factors like woman’s age, environmental conditions, diet, socioeconomic status and psychological parameters, higher doses of nutrients might be required to see any effect on pregnancy outcomes [47].

The current study had a few limitations. The sample size was not large enough to detect more detailed outcomes changes following supplementation. Six weeks of intervention might not be long enough to determine any changes resulted from multi-nutrients supplementation. Therefore, future studies with longer duration of the intervention, and larger sample sizes are needed to confirm our findings. In addition, we did not determine the effects of magnesium-zinc-calcium-vitamin D co-supplementation on all of the pregnancy outcomes including the infant respiratory status and the duration of neonatal intensive care, as well as the gene expression related to inflammation and oxidative stress. We were not able to assess intracellular magnesium concentrations in the current study due to funding limitations. However, TAC levels are evaluated using FRAP assays. Since hemoglobin data were not available in this study, we were not able to adjust our results based on circulating hemoglobin levels. This should be considered in the interpretation of our findings. In addition, we did not evaluate the sensitivity of plasma total nitrite concentrations using Griess method.

## Conclusions

Overall, the findings of this study have demonstrated that magnesium-zinc-calcium-vitamin D co-supplementation for 6 weeks to women with GDM may reduce biomarkers of inflammation and oxidative stress.

## Abbreviations

GDM: Gestational diabetes mellitus
GSH: Total glutathione
hs-CRP: High-sensitivity C-reactive protein
MDA: Malondialdehyde
TAC: Total antioxidant capacity
